# Comprehensive Quantitation Using Two Stable Isotopically Labeled Species and Direct Detection of *N*-Acyl Moiety of Sphingomyelin

**DOI:** 10.1007/s11745-017-4279-5

**Published:** 2017-08-02

**Authors:** Kotaro Hama, Yuko Fujiwara, Hidetsugu Tabata, Hideyo Takahashi, Kazuaki Yokoyama

**Affiliations:** Faculty of Pharmaceutical Sciences, Teikyo University, 2-11-1 Kaga, Itabashi-ku, Tokyo, 173-8605 Japan

**Keywords:** Sphingomyelin, Liquid chromatography-electrospray ionization-tandem mass spectrometry, MS/MS/MS analysis, *N*-Acyl fatty acid

## Abstract

Sphingomyelin (ceramide-phosphocholine, CerPCho) is a common sphingolipid in mammalian cells and is composed of phosphorylcholine and ceramide as polar and hydrophobic components, respectively. In this study, a qualitative liquid chromatography-electrospray ionization tandem mass spectrometry (LC–ESI–MS/MS/MS) analysis is proposed in which CerPCho structures were assigned based on product ion spectra corresponding to sphingosylphosphorylcholine and *N*-acyl moieties. From MS/MS/MS analysis of CerPCho, we observed product ion spectra of the *N*-acyl fatty acids as [RCO_2_]^−^ ions as well as sphingosylphosphorylcholine. A calibration curve for CerPCho was constructed using two stable isotopically labeled CerPCho species and then used to quantify the CerPCho species in HeLa cells as a proof-of-principle study. The present study proposes an accurate method for quantifying and assigning structures to each CerPCho species in crude biologic samples by LC–ESI–MS/MS/MS analysis.

## Introduction

Sphingomyelin (ceramide-phosphocholine, CerPCho) is a common sphingolipid in mammalian cells and especially enriched in plasma membranes. CerPCho is a precursor of sphingosine-1-phosphate (S1P) and ceramides. It is well known that S1P is a lipid mediator that exerts multiple functions such as vasculature development through its cognate G protein-coupled receptors [1]. The clinical importance is further highlighted by the development of the immunosuppressant drug FTY720, which targets S1P receptor (S1P_1_) and is used for treating multiple sclerosis [1]. Ceramides have been suggested to function as intracellular signaling mediators for apoptosis [2]. In addition, ceramides and their derivatives, acylceramides, play essential roles in epidermal barrier homeostasis [3]. Therefore, elucidating CerPCho metabolism is important for elaborating the physiologic and pathologic roles of sphingolipids. CerPCho contains a ceramide and a phosphorylcholine that is linked to the 1-hydroxy group of ceramide; a ceramide is composed of a sphingoid long chain base (LCB) and an *N*-acyl fatty acid. The diversity of the *N*-acyl moiety is produced by six *N*-acyl transferases (EC 2.3.1.24) that catalyze sphinganine acylation [4]. In contrast, an LCB consists mainly of d-*erythro*-sphingosine containing 18 carbons and a double bond (termed d18:1) in mammals, although significant amounts of other LCBs, such as d16:1 and d18:2, have been observed [5, 6]. In addition, d20:1 LCB has been observed in the ganglioside fraction of mouse brain [7]. Thus, the combination of an *N*-acyl moiety and LCB results in a number of ceramide and CerPCho species.

Liquid chromatography linked to electrospray ionization tandem mass spectrometry (LC–ESI–MS/MS) has enabled qualitative and quantitative analysis of phospholipid species [8]. For example, each phosphatidylcholine species is identified by assigning product ion spectra of two fatty acids as well as one or two lysophosphatidylcholine derived from precursor ions of each phosphatidylcholine species in MS/MS analysis [9, 10]. In contrast, the product ion of the *N*-acyl moiety of CerPCho was observed from alkaline metal adduct precursor ions by infusion analysis [11], but is not directly observed in LC–ESI–MS/MS analysis. Therefore, *N*-acyl moieties are deduced by differential analysis between precursor ions and product ions corresponding to LCB in both positive and negative ion modes (Table 1) [12, 13]. Recent advances in LC–ESI–MS/MS techniques have enabled identifying not only the number of carbons and double bonds, but also the location of double bonds of LCB and *N*-acyl moieties of CerPCho (Table 1) [14–17]. However, these methods require several additional devices and/or reagents other than mass spectrometry.

**Table 1:** Comparison of methods for structural assignment of sphingomyelin species

Polarity	Method	References	LCB		*N*-FA	
			Number^a^	Position^a^	Number^a^	Position^a^
Positive	LC–MS/MS	[12]	Sphingosine^b^	✖	✖^e^	✖
	MS/MS + alkaline metal (infusion)	[11]	Sphingosine^b^	✖	✔	✖
	LC–MS/MS + O_3_, e^−^, UV	[14–16]	Sphingosine^b^	✔^d^	✔^d,f^	✔^d, f^
Negative	LC–MS/MS	[13]	SPC^c^	✖	✖^e^	✖
	LC–MS/MS + O_3_, radical	[14, 17]	SPC^c^	✔^d^	✔^d,f^	✔^d, f^
	LC–MS/MS/MS using Q2 and LIT		SPC^c^	✖	Fatty acid [RCO_2_]^−^	✖

^a^ Number: number of carbons and double bonds; position: position of double bond in a sphingoid long chain base (LCB) and an *N*-acyl moiety (*N*-FA)

^b^ Other LCBs such as sphinganine and sphingadienine were also directly detected

^c^ Other LCBs such as sphinganine-1-phosphocholine and sphingadienine-1-phosphocholine were also directly detected

^d^ Multiple fragment ions were produced according to the dissociation between C–C or C=C bonds in LCB and an *N*-FA

^e^ The number of carbons and double bonds of *N*-FA were predicted by differential analysis between precursor ion and LCB ion

^f^ Product ions corresponding to *N*-FA were different between each method for dissociation between C–C or C=C bonds

It is important to employ fully validated analytical methods to quantify each analyte. As the matrix in biologic samples could cause ion suppression (or enhancement) effects, the matrix effect needs to be considered to accurately quantify low abundance analytes in samples. In addition, the quantitative range based on the calibration curve of a representative standard compound that was abundant in tissue samples was not useful for estimating the quantitative range of other molecules present in much smaller amounts. This was because very small amounts of a spiked standard compound were almost negligible compared with endogenous molecules in tissue samples. Thus, it is desirable to construct calibration curves for all analytes in a particular matrix. However, this strategy is not feasible in CerPCho analysis because it is difficult to obtain standards of all compounds in a comprehensive analysis. Furthermore, a very similar biologic sample without CerPCho is not available.

**Fig. 1:**
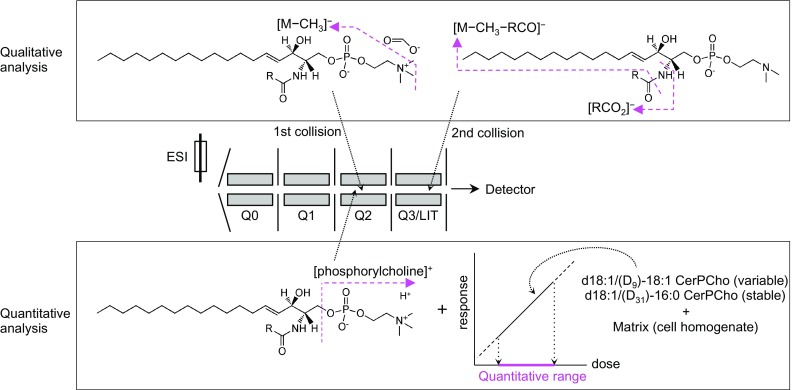
Scheme of CerPCho analysis in this study. The structure of each CerPCho was determined by product ion spectra obtained in negative ion mode. The first precursor ion ([M + HCOO]^−^) was demethylated in a Q2 quadrupole as a collision cell. Product ions from the second precursor ion of [M − CH_3_]^−^ were produced in a Q3/linear ion trap (LIT) and were detected by an electron multiplier. Two product ion spectra corresponding to a sphingosylphosphorylcholine (SPC) and an *N*-acyl moiety were utilized to assign the molecular species of each CerPCho. In contrast, the amount of each CerPCho was quantified by multiple reaction monitoring (MRM) analysis in positive ion mode. Phosphorylcholine ([C_5_H_15_NO_4_P]^+^) dissociated from precursor ion ([M + H]^+^) in Q2 was selectively passed through Q3/LIT and detected. To determine the quantitative range, a calibration curve was constructed using standard samples containing two stable isotopically labeled CerPCho species (d18:1/(D_9_)-18:1 and d18:1/(D_31_)-16:0 CerPCho for a standard and an internal standard compound, respectively) with cell homogenates as biologic matrix

In the present study, we found that the *N*-acyl fatty acid of CerPCho as well as LCB was directly observed as a negative ion in LC–ESI–MS/MS/MS (LC–ESI–MS^3^) analysis (Table 1) without any additional instruments or reagents. In addition, a calibration curve for CerPCho was constructed using two stable isotopically labeled CerPCho species and deteremined the quantitative range for CerPCho species. Based on these observations, we comprehensively qualified and quantified each CerPCho species in HeLa cells by negative ion mode and positive ion mode, respectively (Fig. 1).

## Materials and Methods

### Reagents, Cell Culture and Sample Preparation

Synthesized CerPCho (d18:1/18:1 CerPCho, d18:1/24:0 CerPCho, d18:1/(D_31_)-16:0 CerPCho and d18:1/(D_9_)-18:1 CerPCho) were purchased from Avanti Polar Lipids, Inc. (Alabaster, AL, USA). All chemicals used in mobile phases were purchased from Wako Pure Chemical Industries, Ltd. (Osaka, Japan). HeLa cells (Riken Cell Bank, Riken Bioresource Center, Ibaraki, Japan) were cultured in minimum essential medium (Sigma-Aldrich, Inc., St. Louis, MO, USA) supplemented with 10% fetal bovine serum, 2 mM l-glutamine (Thermo Fisher Scientific Inc., Waltham, MA, USA), 100 U mL^−1^ penicillin and 100 µg mL^−1^ streptomycin (Sigma-Aldrich, Inc.). After washing cells with phosphate-buffered saline three times, cell layers were scraped from the dishes and homogenized in methanol (Wako Pure Chemical Industries, Ltd.) using a vortex mixer and sonication bath. Homogenate protein concentrations were determined using a BCA protein assay kit (Thermo Fisher Scientific, Inc.).

### Plasmids and Transfection

A SpeI-EcoRI fragment of pF1K-human elongation of very long chain fatty acid protein 1 (ELOVL1, EC 6.2.1.3) (FXC20265, Kazusa DNA Research Institute, Chiba, Japan) was transferred into the pcDNA3.1 vector (Thermo Fisher Scientific, Inc.), generating the pcDNA3.1-hELOVL1 plasmid. HeLa cells were transiently transfected with the DNA construct using Lipofectamine 2000 reagent (Thermo Fisher Scientific, Inc.) according to the manufacturer’s instructions and were harvested 72 h after transfection.

### LC–ESI–MS^3^ for Qualitative Analysis

LC–ESI–MS^3^ analysis was performed by modification of a previously described method [18], using a QTRAP4500 (SCIEX, Framingham, MA, USA) linked to a Nexera HPLC system (Shimadzu Corp., Kyoto, Japan). A Capcell Pak C_18_ ACR column (1.5 mm i.d. × 100 mm, particle size 3.0 µm; Shiseido Co., Ltd., Tokyo, Japan) was used at 50°C. The mobile phases were acetonitrile/methanol/water (2/2/1, by vol) with 0.1% formic acid and 0.028% ammonia (A) and isopropanol with 0.1% formic acid and 0.028% ammonia (B). The programmed solvent gradient consisted of solvents A/B at a 100/0 ratio for 5 min, programmed linear alterations to 80/20 over 4 min, to 35/65 over 50 min and to 25/75 over 1 min, after which it was held at 25/75 for 10 min and then linearly to 100/0 over 4 min. The flow rate was 280 µL/min and sample injections 10 µL each. For qualitative analysis, LC–ESI–MS^3^ analysis was performed in negative ion mode, with ions of [M + HCOO]^−^ and [M − CH_3_]^−^ selected as the first and second precursor ions, respectively. Other conditions used in MS^3^ analysis were as follows: ion spray voltage, −4500.0 V; temperature (TEM), 200.0 °C; curtain gas (CUR), 40.0 arbitary units (A.U.); collision gas (CAD), ‘High’; ion nebulizer gas (GS1), 40.0 A.U.; auxiliary gas (GS2), 80.0 A.U.; declustering potential (DP), −26.0 V; entrance potential (EP), −10.0 V; collision energy (CE), −40.0 V; excitation energy (AF2), 0.200 V; scan range, mass to charge ratio (*m*/*z)* 100–1000; scan speed, 10,000 Da/s; fill-time, dynamic; excitation time, 25 ms; quadrupole mass filter (Q1) resolution, ‘unit.’ A 20-µM solution of each synthesized CerPCho species (d18:1/18:1 CerPCho, d18:1/24:0 CerPCho, and d18:1/(D_31_)-16:0 CerPCho) was directly infused into a QTRAP4500 (SCIEX) at a flow rate of 10 µL/min for 30 s to obtain the MS^3^ product ion spectra.

### LC–ESI–MS^3^ for Quantitative Analysis

LC–ESI–MS^3^ for quantitative analysis was conducted using the identical LC conditions as employed for qualitative analysis. Scheduled multiple reaction monitoring (MRM) channels were constructed to cover CerPCho species with 32–52 carbons and 0–7 double bonds present in both LCB and *N*-acyl moieties, in addition to two deuterium-labeled compounds (d18:1/(D_9_)-18:1 CerPCho and d18:1/(D_31_)-16:0 CerPCho), a standard and an internal standard (IS), respectively. Each MRM channel was constructed by selecting protonated molecules ([M + H]^+^) and phosphorylcholine ([C_5_H_15_NO_4_P]^+^) as precursor and product ions, respectively. The time window and cycle time were 360 and 5.4 s, respectively. The following conditions were used in positive ion MRM: ion spray voltage, 5500.0 V; TEM, 300.0 °C; CUR, 40.0 A.U.; CAD, 10.0 A.U.; GS1, 40.0 A.U.; GS2, 80.0 A.U.; Q1 and Q3 linear ion trap (Q3/LIT) resolution, ‘unit;’ DP, 1.0 V; EP, 10.0 V; CE, 35.0 V; collision cell exit potential, 12.0 V. Nitrogen was used as the nebulizer, curtain and collision gas. Analyst software and MultiQuant software (SCIEX) were used for data acquisition and processing. Spectral data were plotted with MjoGraph software (Ochiai Laboratory, Yokohama National University, Japan).

### Accurate Mass Measurement

Accurate masses of product ions from CerPCho were obtained by ion trap/time-of-flight (IT-TOF) MS (Shimadzu Corp., Kyoto, Japan). We directly infused 12.5 nmol of each synthetic CerPCho (d18:1/18:1 CerPCho and d18:1/(D_31_)-16:0 CerPCho) dissolved in acetonitrile/methanol/water/isopropanol (14/14/7/15, by vol) with 0.1% formic acid at 0.05 mL/min with 70% acetonitrile as a mobile phase and the MS/MS product ions were acquired. Precursor ions were detected in the mass range of *m*/*z* 700–1000, with an ion accumulation time of 10 ms (repeat 3). MS/MS fragment ions were acquired in the *m*/*z* 50–800 range under the following conditions: precursor ion isolation width at 1 Da; ion accumulation time of 10 ms; tolerance of 0.05 *m*/*z*; collision-induced dissociation (CID) energy at 50%; execution trigger intensity at 50%. Molecular mass was predicted by the Molecular Mass Calculator at the Biological Magnetic Resonance Data Bank (URL: http://www.bmrb.wisc.edu/metabolomics/mol_mass.php).

### Method Validation

Sample solutions for a spiked calibration curve were prepared as follows. A 100-µM stock solution of d18:1/(D_9_)-18:1 CerPCho in methanol was prepared as a standard compound and diluted further with methanol to prepare standard solutions of 0.1, 0.5, 1, 5, 10 and 50 µM. A 10-µM stock solution of d18:1/(D_31_)-16:0 CerPCho in methanol was also prepared as an IS. Then, 50 µL of IS solution and each diluted standard compound solution was placed into a screw-cap glass tube. After 2 mL of methanol with 1.4 mg of HeLa cell homogenate protein, 1 mL of chloroform (Wako Pure Chemical Industries, Ltd.) and 0.8 mL of water had been added, the total lipid fraction was extracted by the Bligh and Dyer method [19]. The resulting lower organic phase was dried under a nitrogen stream and the residue was solubilized in 500 µL of 99.5% ethanol, followed by filtering through a single-use syringe with a 0.02-µm filter (Millipore Corp., Billerica, MA, USA). Samples were stored at −20 °C until analysis. For validation of the method, three samples with 0.1, 1 and 10 pmol of standard compound per injection were analyzed as quality check compounds (QC). For generating a linear regression curve, 1/*x* ^*2*^ was used as a weighting factor [20]. Accuracy was calculated as [(observed concentration − endogenous concentration)/nominal concentration − 1] × 100(%) and the coefficient of variation evaluated as precision.

## Results

### Assignment of *N*-acyl Moieties in CerPCho

**Fig. 2:**
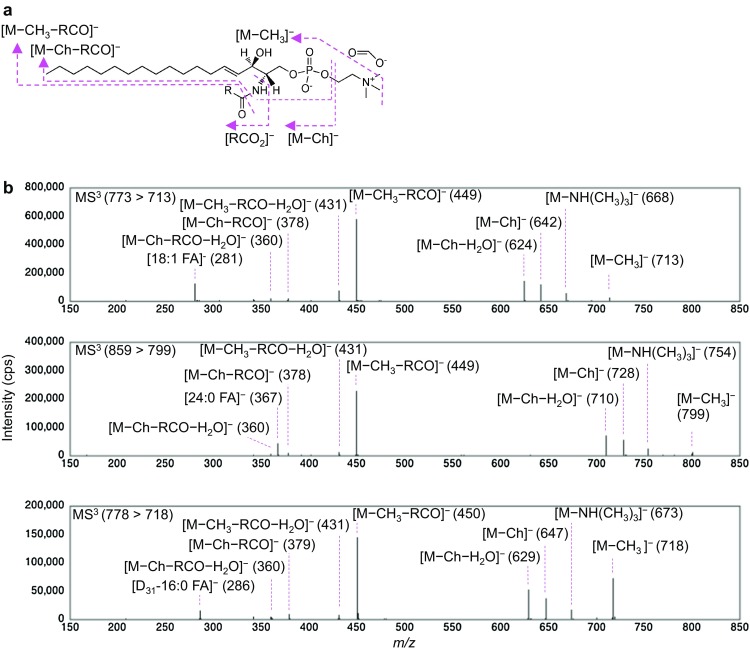
Product ion spectra of CerPCho in MS^3^ analysis. **a** Fragmentation pattern of CerPCho in MS^3^ analysis. **b** MS^3^ spectra derived from d18:1/18:1 CerPCho (*top panel*), d18:1/24:0 CerPCho (*middle panel*) and d18:1/(D_31_)-16:0 CerPCho (*lower panel*) in negative ion mode. A 20-µM solution of each CerPCho in methanol/acetonitrile/water/isopropanol (18/18/9/20, by vol) with 0.1% formic acid and 0.028% ammonia was infused at a rate of 10 µL/min. The *m*/*z* values of the first and second precursor ions analyzed in MS^3^ analysis are indicated in each panel. The choline moiety is represented as “Ch.”

**Fig. 3:**
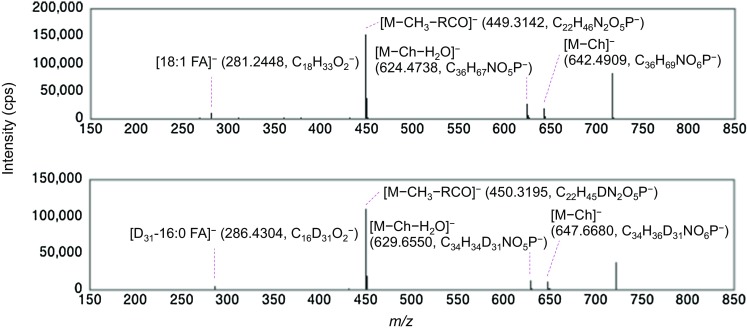
Accurate mass analysis of product ions from CerPCho. 12.5 nmol of d18:1/18:1 CerPCho (*upper panel*) and d18:1/(D_31_)-16:0 CerPCho (*lower panel*) in methanol was infused at 50 µL/min with acetonitrile/water (70/30, by vol) into an IT-TOF MS instrument, and MS/MS spectra derived from a precursor ion ([M − CH_3_]^−^) were obtained. Peaks corresponding to [RCO_2_]^−^ and SPC were observed

Three CerPCho species consisting of the same sphingoid LCB (d18:1) and a variety of *N*-acyl moieties (18:1 fatty acid (FA), 24:0 FA, or (D_31_)-16:0 FA) were analyzed in negative ion mode. First, a Q1 and Q3/LIT were used for separation of the first ([M + HCOO]^−^) and second ([M − CH_3_]^−^) precursor ions, respectively. Second, precursor ions of [M − CH_3_]^−^ were then collided and product ions analyzed in Q3/LIT (Fig. 2a). A significant product ion peak at *m*/*z* of 449 was observed for d18:1/18:1 CerPCho and d18:1/24:0 CerPCho, as has been previously reported [13], while another significant peak was observed at *m*/*z* 450 for d18:1/(D_31_)-16:0 CerPCho (Fig. 2b). Using IT-TOF MS, it was confirmed that the *m*/*z* 449 peak corresponded to sphingosylphosphorylcholine (SPC; C_22_H_46_N_2_O_5_P, predicted and observed *m*/*z*, 449.3144 and 449.3142, respectively; Fig. 3) and that the *m*/*z* 450 peak corresponded to SPC containing one deuterium (C_22_H_45_DN_2_O_5_P, predicted and observed *m*/*z*, 450.3207 and 450.3195, respectively; Fig. 3). Significant peaks at *m*/*z* 281, 367 and 286 were found to be d18:1/18:1 CerPCho, d18:1/24:0 CerPCho and d18:1/(D_31_)-16:0 CerPCho, respectively. A difference (Δ*m*/*z*) of 86 between *m*/*z* 281 and 367 was the same as the molecular mass difference between oleic and lignoceric acids (MW of 282 and 368, respectively; Fig. 2b). It was likely that the Δ*m*/*z* of 81 between *m*/*z* 367 and 286 and Δ*m*/*z* of 5 between *m*/*z* 281 and 286 were the same as the mass differences between lignoceric acid and D_31_-palmitic acid (the latter MW 287) and oleic acid and D_31_-palmitic acid, respectively. The accurate masses of these product ions were examined using IT-TOF MS and the observed significant signal at *m*/*z* of 281.2448 and 286.4304 in d18:1/18:1 CerPCho and d18:1/(D_31_)-16:0 CerPCho, respectively (Fig. 3). As the predicted *m*/*z* for [C_18_H_33_O_2_]^−^ and [C_16_D_31_O_2_]^−^ ions is 281.2481 and 281.4270, respectively, the peaks of *m*/*z* 281 and 286 were concluded to be [C_18_H_33_O_2_]^−^ and [C_16_D_31_O_2_]^−^ ions, respectively. Unexpectedly, the product ions of fatty acids contained two oxygens and not a nitrogen and an oxygen, indicating that the nitrogen in the amide bond was replaced with an oxygen in the collision process. These results showed that the structure of the ceramide moiety in sphingomyelin could be assigned using both product ions of LCB and *N*-acyl fatty acid by LC-ESI-MS^3^ analysis in negative ion mode.

### Calibration Curve and Quantitative Analysis of CerPCho

The method was validated to allow quantitation of each CerPCho species. Two deuterium-labeled CerPCho species were used to construct a spiked calibration curve that was applicable to CerPCho species with very small amounts in biologic samples; d18:1/(D_9_)-18:1 CerPCho and d18:1/(D_31_)-16:0 CerPCho served as a standard compound and IS, respectively. Spiked standard solutions were analyzed using a scheduled MRM mode, and the linearity was examined over a range of 0.1–50 pmol/injection (Table 2). Accuracy and precision values for 0.1, 1 and 10 pmol/injection were within 15%, which was consistent with guidelines for quantitative analysis using MS (Table 2) [21]. These results show that 0.1–50 pmol of CerPCho can be quantified by the present quantitative method using two stable isotopically labeled CerPCho species.

**Table 2:** Calibration curve for sphingomyelin species

Compound	Range (pmol)	Weight	Linearity				Precision (CV(%))^a^			Accuracy (%)		
			Slope	Intercept	*r* ^2^		QC-L^b^	QC-M^b^	QC-H^b^	QC-L^b^	QC-M^b^	QC-H^b^
(D_9_) CerPCho/(D_31_) CerPCho	0.1–50	1/*x* ^2^	0.200	−0.052	0.985	Intra-day (*n* = 12)	3.9	2.2	1.9	3.2	2.3	10.1
						Inter-day (*n* = 4 + 4 + 4)	6.8	6.0	2.0	7.8	4.8	12.3

^a^ Precision was calculated as the coefficient of variation (CV)

^b^ Three samples with 0.1, 1 and 10 pmol of d18:1/(D_9_)-18:1 CerPCho with 10 pmol of d18:1/(D_31_)-16:0 CerPCho per injection were analyzed as QC-L, QC-M and QC-H, respectively

### Qualitative and Quantitative Analysis of CerPCho Species in HeLa Cells

**Fig. 4:**
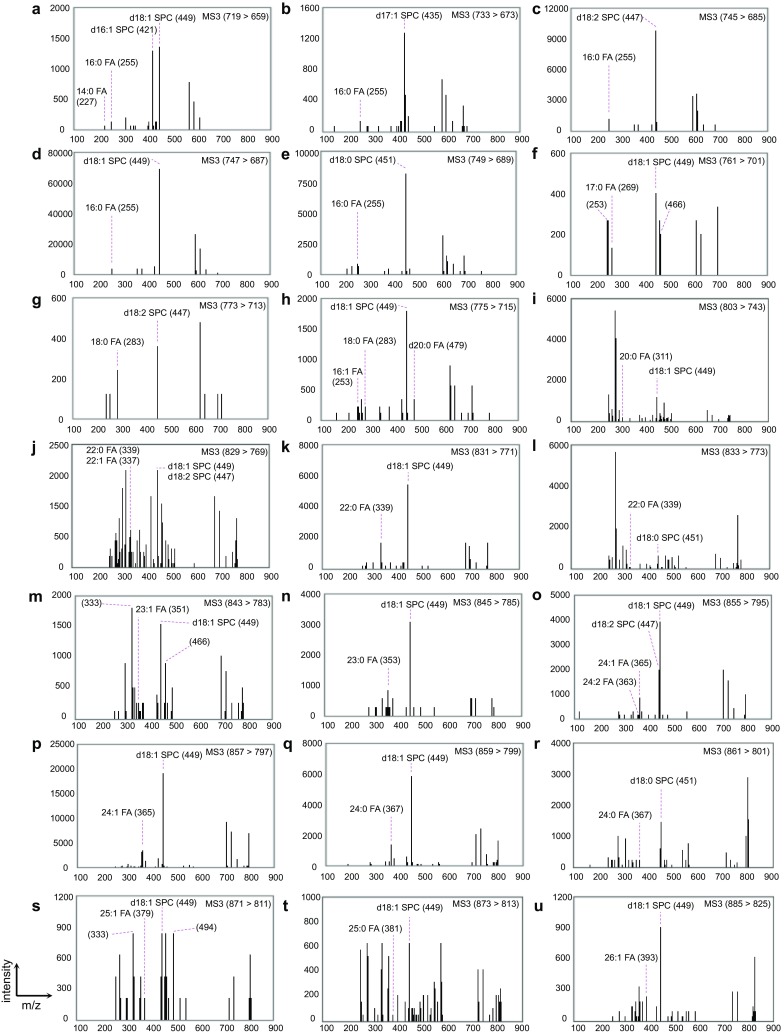
MS^3^ spectra of specific *m*/*z* signals of CerPCho in HeLa cells. Samples extracted from HeLa cells were separated by HPLC equipped with a C_18_ column and analyzed by ESI-MS^3^ employing [M + HCOO]^−^ and [M − CH_3_]^−^ as first and second precursor ions, respectively. Each spectrum corresponding to *N*-acyl moieties and SPC are indicated with *m*/*z*. Each CerPCho species was determined by assigning both product ions of the *N*-acyl moiety and SPC, and they are summarized in Table 3. The *m*/*z* of first and second precursor ions analyzed in MS^3^ analysis are indicated in each panel. Note that the peaks of *m*/*z* 253 and 466 (**f**), 333 and 466 (**m**) or 333 and 494 (**s**) might have been the product ions from coeluted choline plasmalogen species with an isotope. MS^3^ spectral data presented in panel **a**–**h**, **k**, **m**–**q** and **s** were derived from non-treated HeLa cells. The data in **i**, **j**, **l**, **r**, **t** and **u** were from HeLa cells transfected with pcDNA3.1-hELOVL1 plasmid

**Fig. 5:**
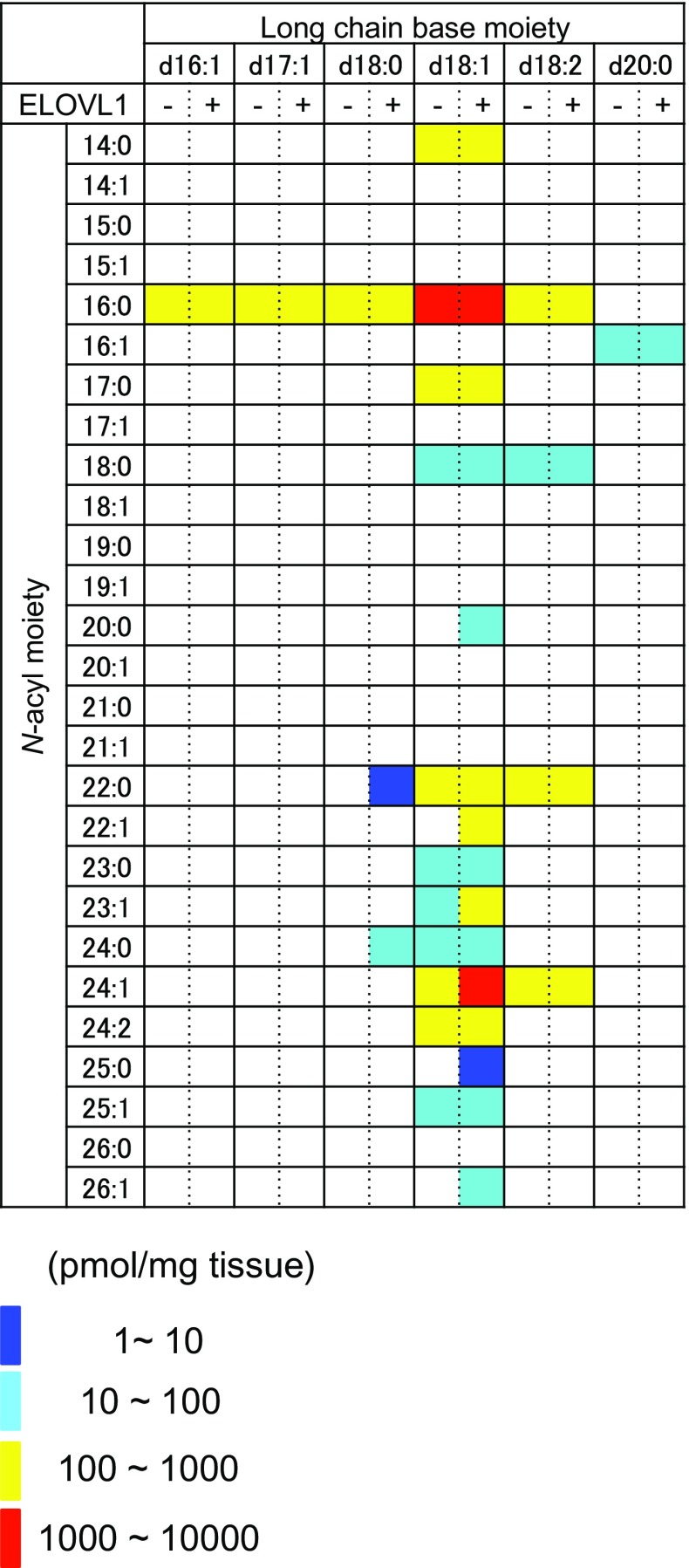
The amount of each sphingomyelin species in non-treated HeLa cells (indicated as ‘−’) or HeLa cells transfected with pcDNA3.1-hELOVL1 plasmid (as ‘+’) is represented on a logarithmic scale (pmol/mg protein). CerPCho species with amounts less than the quantitative range or the species whose structure was not determined in qualitative analysis are indicated in *white*; actual values listed in Table 3

Next, a comprehensive qualitative analysis of CerPCho species in mammalian cells was conducted. Each CerPCho species in a total lipid sample from HeLa cells was resolved on a C_18_ column by HPLC and analyzed by ESI-MS^3^. *N*-acyl moieties were observed with eight different carbon numbers ranging from 14 to 25, including odd numbers (17, 23 and 25; Fig. 4f, m, n, s; Table 3). Most of these *N*-acyl moieties were saturated or monounsaturated. The only polyunsaturated FA observed was 24:2 FA (Fig. 4o; Table 3). In addition, most of the CerPCho species contained LCB with 18 carbons, and LCBs with 16, 17 and 20 carbons were also found (Fig. 4a, b, h; Table 3). Most of the LCB contained zero or one double bond, presumably corresponding to sphinganine and sphingosine, respectively, while two double bonds were observed only in LCB with 18 carbons (Fig. 4c, g, j, o; Table 3). It should be noted that product ions for choline plasmalogen species with an isotope were observed in this analysis. For example, the product ion spectra for 1-*O*-hexadec-1′-enyl-2-*O*-palmitoleoyl-*sn*-glycero-3-phosphocholine, which appeared to contain an isotope, were observed at *m*/*z* 253 (16:1 FA) and 466 (demethylated 1-*O*-hexadec-1′-enyl-*sn*-glycero-3-phosphocholine; Fig. 4f). It was likely that the product ion spectra for 1-*O*-hexadec-1′-enyl-2-*O*-docosatrienoyl-*sn*-glycero-3-phosphocholine and 1-*O*-octadec-1′-enyl-2-*O*-docosatrienoyl-*sn*-glycero-3-phosphocholine, which appeared to contain an isotope, were observed at *m*/*z* 333 (22:3 FA) and 466 (demethylated 1-*O*-hexadec-1′-enyl-*sn*-glycero-3-phosphocholine; Fig. 4m) or 494 (demethylated 1-*O*-octadec-1′-enyl-*sn*-glycero-3-phosphocholine; Fig. 4s). According to the quantitative range, each CerPCho species in HeLa cells was quantified. Assuming the identical extraction and ionization efficiency for each CerPCho species, d18:1/16:0 CerPCho and d18:1/24:1 CerPCho were the most and second most abundant CerPCho species whose structures were determined, and they consist of 54 and 14% of total CerPCho, respectively (Table 3; Fig. 5). The most abundant *N*-acyl moiety was a 16-carbon fatty acid (~70% of total CerPCho) (Table 3; Fig. 5). For the LCB moiety, d18:1 SPC was most abundant (~80%), and d18:2 and d16:1 SPC (14 and ~1%, respectively) were also observed (Table 3; Fig. 5).

**Table 3:** Molecular species of sphingomyelin in HeLa cells

Signals^a^	Amount (pmol/mg protein)		*m*/*z*	Retention time (min)	*N*-FA (*m*/*z*)	SPC (*m*/*z*)	Molecular species	Panel in Fig. 4 ^c^
	Non-treated	ELOVL1^b^						
32:1 CerPCho	115.5	173.2	719	13.3	14:0 (227)	d18:1 (449)	d18:1/14:0	a
					16:0 (255)	d16:1 (421)	d16:1/16:0	a
33:1 CerPCho	239.8	150.7	733	14.7	16:0 (255)	d17:1 (435)	d17:1/16:0	b
34:2 CerPCho	449.9	477.0	745	13.9	16:0 (255)	d18:2 (447)	d18:2/16:0	c
34:1 CerPCho	3355.1	4545.8	747	16.3	16:0 (255)	d18:1 (449)	d18:1/16:0	d
34:0 CerPCho	203.8	342.0	749	17.7	16:0 (255)	d18:0 (451)	d18:0/16:0	e
35:1 CerPCho	106.3	322.6	761	18.4	17:0 (269)	d18:1 (449)	d18:1/17:0	f
36:2 CerPCho	26.5	32.6	773	17.0	18:0 (283)	d18:2 (447)	d18:2/18:0	g
36:1 CerPCho	94.9	76.2	775	19.9	16:1 (253)	d20:0 (479)	d20:0/16:1	h
					18:0 (283)	d18:1 (449)	d18:1/18:0	h
38:1 CerPCho	N.D.^d^	34.9	803	24.0	20:0 (311)	d18:1 (449)	d18:1/20:0	i
40:2 CerPCho	102.3	112.5	829	24.2	22:0 (339)	d18:2 (447)	d18:2/22:0	j
					22:1 (337)	d18:1 (449)	d18:1/22:1^e^	j
40:1 CerPCho	235.0	159.3	831	28.0	22:0 (339)	d18:1 (449)	d18:1/22:0	k
40:0 CerPCho	N.D.^d^	8.0	833	29.7	22:0 (339)	d18:0 (451)	d18:0/22:0	l
41:2 CerPCho	83.3	140.9	843	25.9	23:1 (351)	d18:1 (449)	d18:1/23:1	m
41:1 CerPCho	23.9	31.1	845	30.1	23:0 (353)	d18:1 (449)	d18:1/23:0	n
42:3 CerPCho	286.5	255.1	855	24.6	24:2 (363)	d18:1 (449)	d18:1/24:2	o
					24:1 (365)	d18:2 (447)	d18:2/24:1	o
42:2 CerPCho	853.2	1104.3	857	27.9	24:1 (365)	d18:1 (449)	d18:1/24:1	p
42:1 CerPCho	94.6	20.7	859	32.1	24:0 (367)	d18:1 (449)	d18:1/24:0	q
42:0 CerPCho	N.D.^d^	26.9	861	33.7	24:0 (367)	d18:0 (451)	d18:0/24:0	r
43:2 CerPCho	12.9	24.2	871	29.6	25:1 (379)	d18:1 (449)	d18:1/25:1	s
43:1 CerPCho	N.D.^d^	3.3	873	34.3	25:0 (381)	d18:1 (449)	d18:1/25:0	t
44:2 CerPCho	N.D.^d^	12.3	885	31.9	26:1 (393)	d18:1 (449)	d18:1/26:1	u

^a^ Each sphingomyelin (CerPCho) species observed in quantitative analysis was represented by the total carbon and double bond number of a LCB and an *N*-acyl moiety

^b^ HeLa cells were transfected with pcDNA3.1-hELOVL1 plasmid and cultured in culture medium with 10% FCS. Cells were harvested 72 h after transfection

^c^ Product ion spectra for each CerPCho species were presented in each panel of Fig. 4

^d^ CerPCho species that fragment spectra could not be assigned in the qualitative analysis were referred to as ‘N.D.’

^e^ Product ion spectra for d18:1/22:1 CerPCho were observed in HeLa cells transfected with pcDNA3.1-hELOVL1 plasmid but not in non-treated cells

### Effect of ELOVL1 Overexpression on CerPCho Species in HeLa Cells

ELOVL1 was shown to be important for the production of sphingolipids with 24-carbon fatty acids [22]. We finally examined the effect of ELOVL1 overexpression on CerPCho species comprehensively using the present method. We identified six CerPCho species (d18:1/20:0, d18:0/22:0, d18:1/22:1, d18:0/24:0, d18:1/25:0, and d18:1/26:1) in HeLa cells transfected with pcDNA3.1-hELOVL1 plasmid that were not observed in non-treated cells (Fig. 4i, j, l, r, t, u; Table 3). Quantitative analysis revealed that the total amount of CerPCho species with 26:1 FA, 25:0 FA, 25:1 FA, 24:1 FA, 23:0 FA, 23:1 FA, 22:0 FA or 20:0 FA as *N*-acyl moieties was relatively increased (Fig. 5; Table 3). These results are consistent with the previous result of ELOVL1 knockdown in HeLa cells; ELOVL1 mainly converts 16:0 FA and 18:0 FA into VLCFA [22]. Unexpectedly, the total amount of CerPCho species with 14:0 FA, 16:0 FA or 17:0 FA as *N*-acyl moieties was also increased (Table 3). This result may be partially explained by the sufficient supply of long chain fatty acids from fetal bovine serum in culture medium.

## Discussion

It was critical for structural estimations to properly select product ions from the targeted precursor ion in MS/MS analysis. In positive ion mode, product ions for sphingosine such as sphingoid LCB and *N*-acyl moieties were observed from alkaline metal adduct precursor ions such as [M + Li]^+^ [11]. As crude biologic samples might have contained some salts, depending on extraction methods, alkaline metal adduct ions might have been observed by infusion-ESI-MS/MS analysis. However, the intensities of alkaline metal adduct ions were significantly lower compared with those of protonated ions, at least in the present LC-ESI-MS^3^ analysis (data not shown). This was possibly because alkaline metal ions in samples were mostly eluted in the void fraction. The *N*-acyl moiety of CerPCho has been assigned based on mass differences between precursor and product ions of LCB in LC-ESI-MS/MS analysis [12, 13]. This procedure was simple and feasible; however, the analysis might have been hampered in the case of biologic samples that contained a number of endogenous substances other than the targets. For example, the product ion corresponding to d18:1 SPC might have been wrongly assigned as a 30:1 FA because the *m*/*z*s of both product ions were identical (*m*/*z* 449). The present LC-ESI-MS^3^ analysis increases the signal-to-noise ratio by selecting the precursor ion at Q1 and Q3/LIT and enables observing the product ions of an *N*-acyl moiety and SPC, facilitating proper assignment of CerPCho species. Note that our present method still has some limitations. First, it is still difficult to distinguish CerPCho and other phospholipids that contain a phosphorylcholine as their polar head group, such as in phosphatidylcholine and choline plasmalogen (Fig. 4f, m, s). This was because the molecular ion with anionic adducts and the demethylated molecular ion were usually selected as the first and second precursor ions, respectively, in ESI–LC–MS^3^ analysis under HPLC conditions using conventional mobile phases [13, 18]. Second, the precise structure such as the location of the double bond, isomer (*cis* or *trans*) and shape (straight or branched) of *N*-acyl moieties cannot be determined in the present method.

Intriguingly, the molecular formula of product ions corresponding to *N*-acyl moieties was [RCO_2_]^−^ ions that do not contain nitrogen. Previous studies regarding the CID process for ceramide have proposed that an *N*-acylaminoethanol anion is an intermediate that rearranges into carboxyethylamine, which further dissociates to a [RCO_2_]^−^ ion [23]. In the present study, it was unclear whether a similar rearrangement occurred in the CID process for CerPCho, as the product ion of ceramide was not significantly observed in our MS^3^ analysis (data not shown). Here, product ions of d18:1 SPC from d18:1/(D_31_)-16:0 CerPCho contained a deuterium (Fig. 3), indicating that a deuterium in palmitic acid was incorporated into the LCB moiety in the CID process. Notably, in the previous report, a weak product ion at *m*/*z* of 199 that presumably corresponds to [C_11_H_23_CO_2_]^−^ was observed in MS^3^ analysis of d18:1/12:0 CerPCho using 4000 QTRAP instrument (SCIEX) [24]. Since both 4000 QTRAP and QTRAP4500 used in this study consist of a hybrid triple quadrupole/linear ion trap (LIT), the CID process of CerPCho observed in both studies might be specific to the LIT system. Further study is needed to clarify the CID process for CerPCho.

CerPCho species with 18:1 FA were not observed within the quantitative range in this analysis (Fig. 5). Considering that oleoyl coenzyme-A (CoA) is the most abundant fatty-acyl CoA in HeLa cells (more than tenfold compared with stearoyl-CoA, unpublished data), this result supported the strict substrate specificity of ceramide synthases (EC 2.3.1.24) that have been identified to date [3, 25]. In addition, 16:0 and 24:1 FA were the first and second most abundant *N*-acyl moieties in HeLa cells, respectively, and the amount of CerPCho species with VLCFA is relatively increased by ELOVL1 overexpression (Table 3). These results were in close agreement with previous reports regarding HeLa cells and human plasma [22, 26], showing the feasibility of the present method for the study of CerPCho metabolism. Mammalian LCB biosynthesis is initiated by condensation of *l*-serine with palmitoyl-CoA to generate 3-ketodihydrosphingosine that is catalyzed by serine palmitoyltransferase (SPT, EC 2.3.1.50) [27]. It has been reported that palmitoyl-CoA is the best substrate for this enzyme and that myristoyl and palmitoleoyl-CoA are poor substrates (~15% of substrate activity) [28]. In addition, here, myristoyl-CoA concentrations were 40% that of palmitoyl-CoA, and palmitoleoyl-CoA concentrations were almost the same as those of palmitoyl-CoA in HeLa cells (present unpublished data). Thus, these results indicated that the LCB moiety of CerPCho was mostly determined by substrate specificity and availability of SPT.

In conclusion, CerPCho structures were reliably assigned by product ions corresponding to *N*-acyl moieties as well as LCB in complex biologic samples using the method proposed here. As CerPCho metabolism has been reported to significantly vary during the cell cycle [29] and CerPCho metabolism is altered in several diseases, such as Niemann-Pick disease, this method will be useful for analyzing the metabolism of each CerPCho species. This would allow elucidation of the machinery of sphingoid lipid synthesis and metabolism in physiologic and pathologic conditions.

## Abbreviations

LC–ESI–MS/MS/MS: Liquid chromatography-electrospray ionization-tandem mass spectrometry
MS^3^: MS/MS/MS
CerPCho: Ceramide-phosphocholine/sphingomyelin
SPC: Sphingosylphosphorylcholine
MRM: Multiple reaction monitoring
CID: Collision-induced dissociation
LCB: Sphingoid long chain base
IS: Internal standard
QC: Quality check compounds
